# Effect on vestibular function of cochlear implantation by partial deafness treatment–electro acoustic stimulation (PDT–EAS)

**DOI:** 10.1007/s00405-019-05425-5

**Published:** 2019-05-03

**Authors:** Magdalena Sosna, Grażyna Tacikowska, Katarzyna Pietrasik, Henryk Skarżyński, Artur Lorens, Piotr H. Skarżyński

**Affiliations:** 1 Otorhinolaryngosurgery Clinic, Institute of Physiology and Pathology of Hearing, Warsaw, Poland; 2 Department of Otoneurology, Institute of Physiology and Pathology of Hearing, Warsaw, Poland; 3 Department of Auditory Implant and Perception, Institute of Physiology and Pathology of Hearing, Warsaw, Poland; 4 World Hearing Center, Institute of Physiology and Pathology of Hearing, Mokra 17 Street, 05-830 Kajetany, Poland; 5 Institute of Sensory Organs, Kajetany, Poland; 6 Heart Failure and Cardiac Rehabilitation Department, Medical University of Warsaw, Warsaw, Poland

**Keywords:** Partial deafness treatment, Cochlear implantation, Balance, Vestibule, Atraumatic, Electro-acoustic stimulation

## Abstract

**Purpose:**

Although the cochlear implantation procedure does not interfere with vestibular structures directly, both the vestibulum and the cochlea share the same inner ear fluid space, and this fluid may be responsible for transferring possibly damaging forces from one to the other. The purpose of the study is to assess postoperative vestibular function after partial deafness treatment–electro-acoustic stimulation (PDT–EAS) cochlear implantation.

**Methods:**

Fifty-five patients were included in the study (30 females, 25 males, age 11–80, mean 41.8 ± 19.35). cVEMP and oVEMP were performed preoperatively and 1–3 months after cochlear implantation. Caloric and vHIT tests were conducted preoperatively and 4–6 months after cochlear implantation.

**Results:**

Our study shows that, based on a wide range of electrodes, use of PDT–EAS is protective in terms of preserving vestibular function. It gives a rate of saccular damage of 15.79%, utricular damage of 19.04%, and a horizontal semicircular canal response reduction of 15.79%.

**Conclusions:**

PDT–EAS is protective in terms of preserving vestibular function. Nevertheless, it should be emphasized that the risk of vestibular damage cannot be totally eliminated even when hearing preservation techniques are adopted.

## Introduction

Vestibular and balance disorders are common complaints reported by patients after cochlear implantation (CI) [1–3]. Although the CI procedure does not interfere with vestibular structures directly, both the vestibulum and the cochlea share the same inner ear fluid space, and this fluid may be responsible for transferring possibly damaging forces from one to the other. Of course, there are multiple factors involved, and the reason why cochlear implantation appears to have effects on the vestibular organ is still a matter for further research [4]. The most plausible factors causing postoperative vertigo are labyrinthine irritation and inflammation from foreign bodies (blood, bone dust, electrode), a reaction called serous labyrinthitis [1, 3], intraoperative perilymph loss [5], electrode insertion trauma that may cause direct damage to hair cells or their necrosis due to the mixing of endolymph and perilymph when the basilar membrane ruptures [6–8] Moreover, there are cases where otoconia have been apparently dislodged as a result of intraoperative drilling or electric current spread during CI activation, with consequent benign paroxysmal positional vertigo [9]. Less directly, after the implantation, other persistent vestibular conditions may arise such as fibrosis and obliteration of the inner ear, endolymphatic hydrops caused by the disturbance of inner ear fluid homeostasis [7], and electric co-stimulation of vestibular fibers [10].

Because the indications for cochlear implantation have steadily broadened and now include cases with residual low-frequency hearing, unilateral deafness, and bilateral implantation, this places even greater emphasis on protecting not only the cochlea, but also the vestibular structures.

Many papers have already been devoted to the assessment of vestibular function after cochlear implantation using either cochleostomy or the round window approach. They report a big discrepancy in the incidence of postoperative vestibular tests deterioration: 31.25–86.00% cVEMPs, 6.25–50.00% caloric tests in cochleostomy and 0.00–76.47% cVEMPs, 4.70–36.84% oVEMPs, 0.00–93.10% caloric tests in round window approach [11–23].

**Fig. 1:**
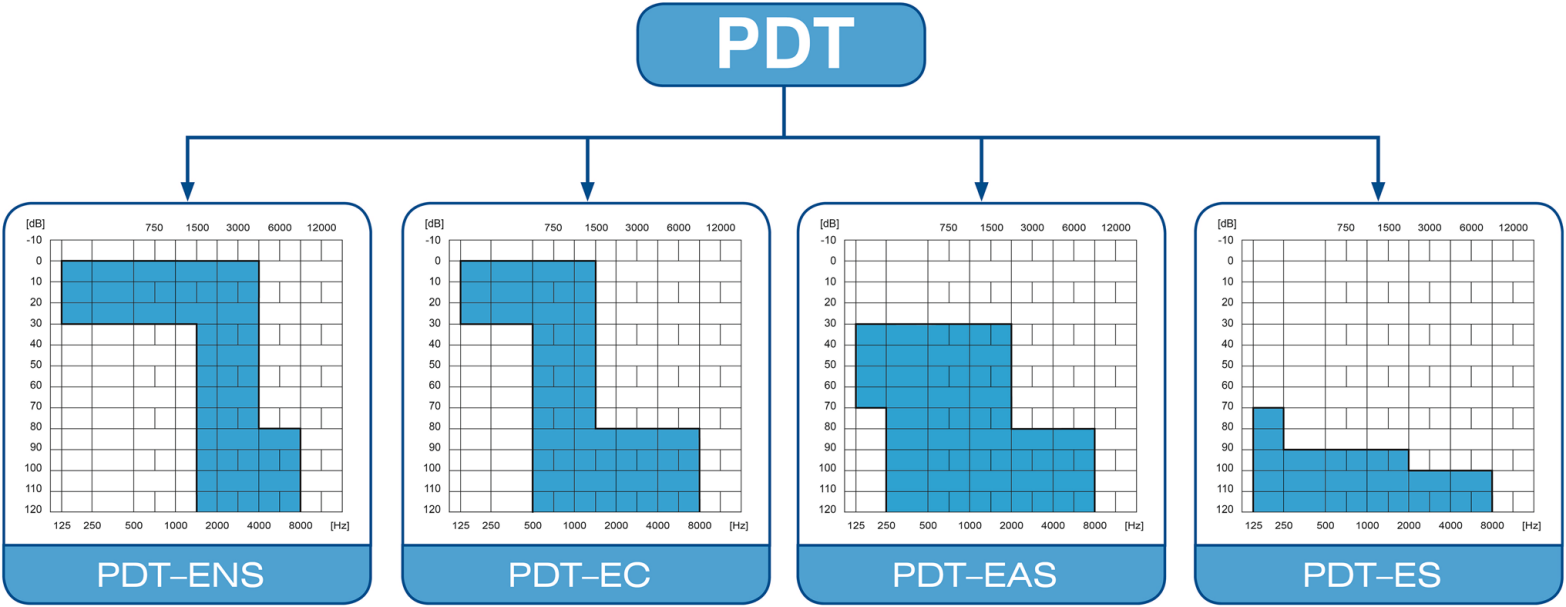
Four broad categories of partial hearing loss and how each is suited to four different types of partial deafness treatment

What is more, only a few papers have addressed the diversity of cochlear implantation procedures in partial deafness treatment where the patient is not absolutely deaf, but still has appreciable levels of residual hearing. As shown in Fig. 1, partial deafness treatment can be divided into the following groups: electro-natural stimulation (PDT-ENS)—patients with normal or only slightly elevated thresholds in low- and mid-frequency bands, who need electrical complementation with a very short electrode; electrical complement (PDT-EC)—patients with normal or only slightly elevated thresholds at low frequencies, who need electrical complementation with short electrodes and no amplification at the apical region; electro-acoustic stimulation (PDT–EAS)—patients with low- and mid-frequency residual hearing who need amplification from a hearing-aid for low frequencies and electric stimulation from implanted electrode for mid and high frequencies; and electrical stimulation (PDT-ES)—includes patients with non-functional residual hearing [24–28].

Of these four, this paper is concerned with the third, partial deafness treatment–electro-acoustic stimulation (PDT–EAS), the most frequent situation in partial deafness. It claims to be successful in terms of hearing preservation and gives satisfactory speech discrimination [29–34], but only a single article has focused on this group in terms of postoperative vestibular function.

Different surgical strategies have been demonstrated in the literature to be adequate for PDT–EAS [29–34]. We have found the following aspects to be crucial. Insertion of the electrode via the round window lowers the risk of osseous spiral lamina destruction or electrode misinsertion into the vestibule, since the electrode projects directly into the scala tympani. Use of soft electrodes also reduces electrode insertion trauma. Reducing the insertion angle makes it less likely that any cochlear structure will be damaged, since the cochlea is thicker in its basal region [35–37]. Introducing only a micropuncture of the round window decreases intraoperative perilymph loss and the risk of disturbing otoconia. Administering steroids after the operation is important as they have an anti-inflammatory effect on the inner ear, reducing reaction to foreign bodies and, by absorbing water, helping to avoid endolymphatic hydrops. The electrode inserted in PDT–EAS should provide the cochlea with sufficient electrical stimulation and satisfactory speech perception (even if deterioration in residual hearing occurs later), but should not traumatize the apical region that will be acoustically amplified. The decision of which electrode to use depends on the experience of each clinic, cochlear size, and the stability of hearing loss before cochlear implantation [29–34]. In our clinic, the choice is mostly electrodes 24–28mm long [24, 29].

The aim of this study was to assess the safety of PDT–EAS cochlear implantation in terms of vestibular preservation after insertion of a range of electrodes types.

## Material and methods

Fifty-five patients operated on in the otorhinolaryngosurgery clinic were included in the study (30 females, 25 males, age 11–80, mean 421.8 ± 19.35). The etiology of hearing loss is given in Table 1. Exclusion criteria were as follows: reimplantation cases, superior semicircular canal dehiscence syndrome due to possible false positive vestibular evoked myogenic potential (VEMP) responses and complete vestibular damage before cochlear implantation [indicated by absent cervical VEMP (cVEMP), ocular VEMP (oVEMP) responses, areflexia in caloric test slow-component velocities (SCV) < 5°], and covert or overt saccades in all semicircular canals with the video head impulse test (vHIT). The study was approved by the local ethics committee, and informed consent was obtained from all patients.

**Table 1:** Etiology of deafness

Etiology of deafness	Participant number
Sudden deafness	9
Unknown	27
Viral infection	3
Ototoxic medication	5
Postinflammatory	3
Acoustic trauma	1
Head trauma	2
Genetic	1
Meniere’s disease	1
Meningitis, cholesteatoma	1
TORCH	1
Otosclerosis	1

The implanted ear was the right in 28 patients and the left in 27. In eight cases the patients have been previously implanted and received the second implant on the opposite side. All patients had low-frequency residual hearing and were qualified for the PDT–EAS procedure (pure-tone audiometry threshold > 30 dBHL at 500 Hz, ≤ 70 dBHL at 125Hz, 250 Hz and > 80 dBHL at 4000 Hz). The cochlear implantation followed the following steps: (1) antrotomy; (2) posterior tympanotomy to allow for visualization of the round window niche; (3) puncture of the round window membrane; (4) insertion of the electrode array, approaching the scala tympani directly through the round window membrane; (5) electrode fixation in the round window niche with fibrin glue (with the membrane partially uncovered to preserve its mobility); (6) fixation of the device in a well created in the temporal bone [29]. All patients, apart from three cases, were implanted with soft lateral wall electrodes. The duration of hearing impairment ranged from 1.92 to 55.67 years (*M* = 20.13 years, SD =  ± 13.31 years). The etiological factors and numbers of each different electrode type are shown in Tables 1 and 2.

**Table 2:** Different types of inserted electrodes

Electrode	Participant number
Med-El Sonata Medium	3
Med-El Concerto Medium	1
Med-El Sonata Flex 28	16
Med-El Concerto Flex 28	4
Med-El Synchrony Flex 28	2
Med-El Sonata Flex 24	9
Med-El Concerto Flex 24	3
Med-El Synchrony Flex 24	2
Advanced Bionics Hi-Res 90k Advantage Mid-scala	3
Cochlear Nucleus CI422 Slim straight electrode	1
Cochlear Nucleus CI522 Slim straight electrode	2
Med-El Sonata Flex soft	5
Med-El Concerto Flex soft	2
Med-El Sonata Compressed	1
Med-El Sonata Form 24	1

### VEMP

cVEMP and oVEMP were performed preoperatively and 1–3 months after cochlear implantation using the Eclipse Interacoustics A/S apparatus.

### cVEMP

Stimulation was provided monaurally through insert tips with a 500-Hz tone burst at 97 dBnHL and stimulation rate of 5.1 Hz and stimulation gate 2:2:2. A set of 200 stimuli were averaged. The patient was seated and asked to rotate their head 45° away from the stimulated ear to achieve constant tonic contraction of the sternocleidomastoid (SCM) muscle. An SCM contraction level of 50–150 µV was maintained during the whole examination using visual biofeedback derived from the software. The two active electrodes were placed at the midpoint between the termination of the muscle at the mastoid and its origin at the sternum, the inverting electrode was placed between the sternoclavicular joints, and the ground electrode was attached to the forehead. The impedance of the electrodes was maintained below 2.5 kΩ. The response was regarded as present if two repeatable electromyographic patterns were elicited. The test was performed with the cochlear implant switched off.

The presence of a response, the P1,N1 latency and the amplitude asymmetry ratio were measured (normal range < 36%). The amplitude was corrected by dividing the (P1–N1) amplitude by the prestimulus SCM contraction level.

### oVEMP

oVEMPs were measured using a 500-Hz tone burst at 97 dBnHL, 2:2:2 stimulation gate, stimulation rate of 5.1/s, signal averaging 500 × , and filter band from 0.01 to 1 kHz. The active electrodes were placed infraorbitally in the midline of the eye, reference electrode on the chin, and ground electrode on the forehead. The response was recorded contralaterally and the impedance of the electrode was maintained below 2.5 kΩ. The patient was seated and asked to gaze 35° vertically during the recording. The response was regarded as present if two repeatable patterns were recorded. The test was performed with the cochlear implant switched off.

The presence of a response, N1 latency, interaural amplitude ratio (normal range, 33%), and (P1–N1) amplitude were analyzed.

### Caloric test

We used Visual Eyes VNG of Micromedical Technologies and performed Fitzgerald–Hallpike bithermal caloric stimulation, with two stimulations (44 °C and 30 °C) for 30 s. The second test was performed after a rest of at least 8 min from the first. The patients were supine with a 30° elevation of the upper body. Unilateral weakness (UW) and slow component velocity (SCV) on both sides before and after cochlear implantation were compared. The degree of canal paresis (UW) was calculated based on Jongkees’ formula. A difference of UW > 25% between pre- and postoperative measurements was judged as a weakened response. The examination was conducted before and 4–6 months after cochlear implantation.

### Video head impulse test

vHIT was performed using ICS Impulse type 1085, GN Otometrics. The patient was seated and asked to keep staring at the spot. Then the abrupt, unpredictable, small angle (about 10°–20°) head movements were done in three plains: horizontal, LARP (left anterior–right posterior plane) and RALP (right anterior–left posterior plane). In every case, 20 impulses were delivered with the minimal peak head velocity of 150º/s. Normal gain (the quotient of head movements speed and eye movements speed) ranged within 0.6–1.2. The decrease of gain below 0,6 or the new appearance of covert- or overt saccade was treated as the damage of the particular semicircular canal. The test was conducted preoperatively and 4–6 months postoperatively [7].

### Statistical analysis

A Mann–Whitney *U *test was used to assess the difference in terms of age and duration of hearing loss between patients with preserved and lost vestibular function. A Chi-square test was used to assess the relationship between sex and the vestibular postoperative status. A paired-samples *t* test was applied to assess the parameters of the VEMP and caloric tests before and after cochlear implantation. Statistical analysis was performed using IBM SPSS Statistics v.24. A *p *level < 0.05 was considered as statistically significant.

## Results

### cVEMP

Of the 55 patients included in the study, 38 presented cVEMP responses preoperatively on the operated side. There were 31 patients who showed a bilaterally present VEMP (22 with an amplitude asymmetry ratio within the normal range, 6 with hypofunction of the sacculus on the non-operated ear, 3 with the hypofunction on the operated side) and 7 with VEMP response on the operated side only; the mean latency P1 was 16.51 ms ( ± 1.65 ms), N1 25.25 ms ( ± 1.86 ms). Postoperatively, we noticed a loss of sacculus response in 6 out of 38 patients (15.79%). The mean latency postoperatively was *P*1 = 15.96 ms ( ± 1.67 ms), *N*1 = 24.07 ms ( ± 1.77 ms); the difference between pre- and postoperative VEMP latency was not statistically significant (*p*1 = 0.477, *p*2 = 0.730). The mean age of the patients who lost their cVEMP responses was 55.93 ( ± 11.09), while the mean age of the cochlear implant recipients who maintained sacculus responses was 33.29 ( ± 16.66). According to Mann–Whitney *U* test, the difference between the two groups (with loss and present responses postoperatively) in terms of age was statistically significant *U* = 28.00; *p* = 0.005. The patients who lost their saccular function were implanted with the following electrodes: Flex 24 (*n* = 2), Flex 28 (*n* = 1), Flex soft (*n* = 1), Medium (*n* = 1), CI522 (*n* = 1); the cause of hearing loss was: unknown (*n* = 2), sudden deafness (*n* = 2), viral infection (*n* = 1), head trauma (*n* = 1). The sex distribution (female:male ratio) in the group with maintained and lost cVEMP responses was 16:16 and 5:1, respectively. Although there seemed to be a preponderance of females, it was not statistically significant (*χ*^2^ = 2.27, *p* = 0.132). The mean duration of hearing loss in the group with (18.96 ± 11.57years) and without (16.76 ± 13.08 years) preserved cVEMP after CI did not differ significantly between the two groups (*U* = 84.00; *p* = 0.631).

### oVEMP

We generated oVEMP in 21 out of 55 patients on the operated side. Bilaterally evoked responses were noticed in 17 patients (11 with an amplitude asymmetry ratio within the normal range, 3 with an asymmetry toward the non-operated ear and 3 with asymmetry toward the operated ear) and one-sided responses were elicited in 4 patients preoperatively. We saw no measurable VEMP response in 4 out of 22 patients postoperatively (19.04%). Mean latency before cochlear implantation was *N*1 = 12.39 ms ( ± 1.10ms), *P*1 = 17.5ms ( ± 0.78ms), postoperatively *N*1 = 12.73 ms ( ± 0.84 ms), *P*1 = 17.88 ms ( ± 0.89 ms); the difference was not statistically significant (*p*1 = 0.172, *p*2 = 0.161). Patients who lost their oVEMP responses were older than those who still had elicitable oVEMP after cochlear implantation (mean age 51.35 ± 11,74 and 33.49 ± 15.72, respectively), but the difference was not statistically significant using Mann–Whitney *U* test: *U* = 14.50; *p* = 0.081. The patients who lost their oVEMP response were implanted with the following electrodes: Flex 24 (*n* = 2), Flex 28 (*n* = 1), and CI522 (*n* = 1); and the etiological factors of their hearing loss were: unknown (*n* = 1), sudden deafness (*n* = 2), and head trauma (*n* = 1). The postoperative loss of oVEMP response did not correlate with sex (female:male ratio 8:9 and 3:1 in the groups with maintained and lost utricle responses. respectively;* χ*^2=^1.01, *p* = 0.314) nor with the duration of hearing loss (22.05 ± 15.00 years with maintained and 17.85 ± 16.31 years with lost utricle response; *U* = 27.00; *p* = 0.531).

### Caloric test

Nineteen patients were examined by caloric test before and after cochlear implantation. A significant reduction of caloric response (change of UW > 25% toward the operated side) was found in 3 out of 19 patients (15.79%). Two patient who had reduced excitability of the horizontal semicircular canal did not have a preoperative VEMP response to compare with the caloric response, and one lost his cVEMP response postoperatively as well, but had still present oVEMP response. Mean preoperative SCV was 53.00°/s compared to 42.80°/s postoperatively. The difference was statistically significant (*p* = 0.010). Patients with significant deterioration of caloric response were older (mean age 58.72 ± 6.08) than those with relatively unchanged caloric test postoperatively (mean age 44.11 ± 22.14); however, the difference was not statistically significant according to *U* Mann–Whitney test: *U* = 13.00; *p* = 0.219. The patients with reduced caloric response after CI were implanted with: Flex 24 (*n* = 1), Flex 28 (*n* = 2), and CI522 (*n* = 1). The etiological factors of their hearing loss were: unknown (*n* = 2) and sudden deafness (*n* = 1). There were no statistically significant differences regarding sex distribution (female:male ratio) in the group with preserved and damaged lateral semicircular canal function (7:9 and 3:0, respectively; *χ*^2^ = 3.20, *p* = 0.730) The mean duration of hearing loss did not correlate with postoperative change in caloric response (22.87 ± 13.78 in the group with preserved and 31.05 ± 26.90 in the group with damaged lateral semicircular canal; *U* = 20.00; *p* = 0.655).

### vHIT

vHIT was performed pre- and postoperatively in nine patients. The mean vHIT gain before the cochlear implantation was 1.03 ( ± 0.16) and 0.89 ( ± 0.11) after the cochlear implantation. The difference was statistically significant (p = 0.005). We did not notice any gain < 0,6 in any semicircular canal and any new overt or covert saccade postoperatively. The mean vHIT gain before and after cochlear implantation was, respectively, 1.06 ( ± 0.10) and 0.99 ( ± 0.09) (*p* = 0.045) in the lateral semicircular, 1.06 ( ± 0.19) and 0,87 ( ± 0.12) (*p* = 0.005) in the anterior semicircular canals, and 1 ( ± 0.28) and 0.83 ( ± 0.21) (*p* = 0.010) in the posterior semicircular canal. The summarize of the results both in group with and without maintained vestibular responses is depicted in Table 3.

**Table 3:** Comparison of the groups with and without preserved vestibular responses after PDT–EAS cochlear implantation

=>	cVEMP			oVEMP			Caloric test		
	Preserved	Damaged	Statistical significance	Preserved	Damaged	Statistical significance	Preserved	Damaged	Statistical significance
	32/28 (84.21%)	6/38 (15.79%)		18/22 (80.96%)	4/22 (19.04%)		16/19 (84.21%)	16/19 (84.21%)	
Age	33.29 ( ± 16.66)	55.93 ( ± 11.09)	*U* = 28.00; *p* = 0.005	33.49 (± 15.72)	51.35 (± 11.74)	*U* = 14.50; *p* = 0.081	44.12 (± 22.14)	58.72 (± 0.08)	*U* = 13.00; *p* = 0.219
Sex (female:male ratio)	16:16	5:1	*p* = 0.197	8:9	3:1	*p* = 0.585	7:9	3:0	*p* = 0.211
Duration of hearing loss	16.76 (± 13.08)	18.96 (± 11.57)	*U* = 84.00; *p* = 0.63	22.05 (± 15.00)	17.85 (± 16.32)	*U* = 27.00; *p* = 0.531	22.87 (± 13.78)	31.06 (± 26.90)	*U* = 20.00; *p* = 0.655

## Discussion

Much research has been done comparing vestibular function after cochlear implantation surgery, looking for differences in surgical techniques and approaches (particularly, cochleostomy versus the round window approach) [11–23].

The review of the literature actually does not give a straightforward answer to which access route is better for vestibular preservation: cochleostomy or the round window approach.

The results of vestibular preservation differ significantly among clinics and depend on the surgeon’s experience, technique, type of inserted electrode, and criteria applied for analyzing and comparing the otoneurological tests (cVEMP, oVEMP, caloric responses, vHIT) pre- and postoperatively. While the assessment of hearing preservation is based on strict audiological testing, the comparison of otoneurological tests may be more challenging. Some analyses acknowledge a loss of VEMP as an indicator for saccular or utricle damage, while others point to a reduced VEMP amplitude or elevated threshold for eliciting VEMPs as a marker of otolith hypofunction. Comparisons of caloric tests generally lack uniformity as well. In some cases, the change in unilateral weakness is described as significant, while in other cases any reduction in slow phase velocity on the implanted side is taken to indicate semicircular lateral canal injury after cochlear implantation. A description of the incidence of vestibular damage via the RWA surgical techniques (not limited do PDT–EAS procedure) is summarized in Table 4.

**Table 4:** The prevalence of vestibular damage in the round window approach [15, 17–23]

	Loss of cVEMP response	Loss of oVEMP response/SVV	Reduction of caloric response	Gain reduction in vHIT	Degree of hearing loss	Electrode	Time of examination (after cochlear implantation procedure)
Chen et al. [17]	41.67% (10/24)	36.84% (7/19)	93.10% (27/29)^a^	–	Severe to profound	Not mentioned	4 weeks
Nordfalk et al. [18]	46.15% (12/26)	25.92% (7/27)^b^	36.36% (8/22)^c^	–	Low-frequency residual hearing ≤ 70dBHL 125Hz, 250Hz ≤ 90dBHL 500Hz	Flex 24, Flex 28, Flex soft	6–8 weeks
Meli et al. [19]	76.47% (13/17)	–	12.00% (3/25)^d^	–	Severe to profound	CI24RE, Med-el Concerto (electrode not given), Mid-scala	2 months
Robard et al. [20]	54.54% (12/22)^e^	–	72.40% (21/29)^f^	–	Not given	Contour advance, Hybrid L24, CI422	5 months
Louza et al. [21]	62.00% (18/29)	–	27.00% (8/30)^g^	–	Not given	CI24 RECA, CI24 REST, Flex 28, Flex soft, Standard	4–6 weeks
Tsukada et al. [22]	0.00% (0/11) 9.00% (1/11)^h^	–	0.00% (0/11)	–	Low-frequency residual hearing ≤ 65dB HL 125,250, 500Hz ≥ 80dB HL 2kHz ≥ 85dB HL > 4kHz	Flex 24	Minimal 4 weeks
Rah et al. [15]	–	–	0.00% (0/9)	–	Severe to profound	Not mentioned	12 months
Dagkiran et al. [23]	11.90% (5/42)	4.70% (2/42)	–	2,30% (1/42)^i^	Severe to profound	Medium, slim straight	3 months

*cVEMP* cervical myogenic vestibular potential, *oVEMP* ocular myogenic vestibular potential, *vHIT* video head impulse test, *UW* unilateral weakness in caloric test, *SVV* subjective visual vertical, *SPV* slow phase velocity in caloric test

^a^SPV reduction

^b^Pathological SVV (deviation more than 3°)

^c^>25% change in UW

^d^UW

^e^Loss or reduction of the amplitude in VEM

^f^UW or increasing in already existing deficit

^g^Loss or reduction of SCV

^h^Reduction of VEMP amplitude

^i^Apart from anterior semicircular canal

Even less is known about the impact of insertion depth or electrode length on postoperative vestibular function. Nordfalk et al. [18] measured a loss of VEMP responses in 5 out of 14 patients (35.7%) and weakened caloric response in 4 out of 10 patients (40%) implanted with a Flex28 electrode via a round window approach, but, due to the small number of patients, they did not discuss the results of inserting the shorter electrodes. Louza and colleagues [21] did not find any statistically relevant relationship between postoperative vestibular function and the insertion depth of the electrode (276º–707º).

Tsukada et al. [22] reported no loss of cVEMP and significant reduction in only 1 of 11 patients (9%) after using a round window approach and a Flex EAS (Flex 24 mm) electrode.

Histological studies have found that vestibular damage is significantly reduced when the electrode is inserted into scala tympani [7, 38]. Reduced trauma leads to a decrease in soft tissue reaction and long-term histopathological changes in the cochlea [39].

PDT–EAS implantation involves applying a ‘soft surgery’: the use of a round window approach, postoperative steroid administration, and micropuncture of the round window membrane. However, there are some factors indicating that PDT–EAS cochlear implantation may traumatize the inner ear.

Temporal bone studies have shown that the height at the central and lateral portion of the scala tympani decreases with increasing distance from the round window, whereas the height of the modiolar area remained nearly constant. The height at the lateral wall is reduced significantly after 450°. It increases the risk of unwanted contact of the electrode and basilar membrane, spiral ligament or the osseous spiral lamina and consequently the risk of intracochlear trauma. Also, the mechanical properties of the basilar membrane are different depending on the distances from the round window, while the thickness of this structure decreases toward the apex (although it may differ between individuals). As a result, the rupture force of the basilar membrane differs for each electrode type and insertion depth [35–37].

De Seta et al. found while testing the Flex28 on cadaveric model that the insertion force increased significantly as a function of depth of insertion both with traumatic and atraumatic insertions. The maximal peak forces occurred at the end of the insertion as a result of the friction between the entire array and the lateral cochlear wall and inner ear structures [40].

According to Adunka et al., the insertion forces increased dramatically when the electrode (in this case a Flex soft) was pushed beyond 18–20 mm [41]. All this facts show that in case of deeper lateral wall electrode insertion (which may occur in 24–28 mm long electrodes), intracochlear trauma is more likely to occur. The flex electrodes that we used for deeper electrode insertion in PDT–EAS have some special features that help to avoid intracochlear trauma. The five most apical electrode contacts are single, whereas the basal seven electrodes are paired which reduces the diameter of the electrode tip.

According to some other temporal bone research, the occurrence of severe injuries at a location approximately 150°–180° from the round window is regarded a typical pattern (ascending part of the basal turn, narrowing of the bony capsule, shifting or rotation of the spiral osseous lamina).[36]

Undoubtedly, more studies need to be carried out to investigate the effect of hearing preservation techniques using short electrodes and insertion angle less than 360° (in our classification PDT-EC or even PDT-ENS implantation).

Our study has shown that, based on a wide range of electrodes, use of PDT–EAS is protective in terms of preserving vestibular function. It gives a rate of saccular damage of 15.79%, utricular damage of 19,04%, and a horizontal semicircular canal response reduction of 15.78%. Surprisingly, we noticed no vestibular loss in cases of perimodiolar electrode insertion (*n* = 3), even though they might be expected to be more traumatic for the inner ear due to their stiffness. However, we prefer the use of lateral wall soft electrodes in PDT–EAS and that was the case in all the other patients. We did not notice any effect of electrode types on postoperative vestibular outcome, and both groups of patients were implanted with various types of these.

Our results suggest that age predisposes the patients to postoperative vestibular loss after cochlear implantation. The correlation with age was clearly evident in the case of cVEMPs, visible but not statistically significant in oVEMPs and caloric tests. Up to now, there are many papers showing better hearing preservation in younger adults and adolescents [33, 42], but not in case of vestibular preservation. This finding is important as among CI candidates we now see an increasing numbers of elderly patients who are expected to have less effective central compensation mechanisms should they suffer vestibular damage.

The limitations of the study stem from the small number of patients, especially those who lost their vestibular responses after cochlear implantation. This factor restricts the statistical power of being able to see the impact of particular electrodes and other factors on postoperative vestibular function.

## Conclusions

It should be emphasized that the risk of vestibular damage can be decreased, but never totally eliminated, even when hearing preservation techniques are adopted. That is why special care and counselling are recommended when qualifying a patient for implantation when the ear has the only (or better) vestibulum, since there is then the risk of bilateral hypofunction or areflexia. Special attention should be also paid to elderly patients, as the risk of postoperative loss in vestibular function increase with age and additionally the central nervous compensation mechanism may be slower and less effective.
